# Supplementary material for: Predicting the remaining useful life of metro pantograph sliding strips using gamma processes and its implications for maintenance scheduling
Source: PLoS One. 2025 Jul 11;20(7):e0327769. doi: 10.1371/journal.pone.0327769 (PMC12250525; doi:10.1371/journal.pone.0327769)
Supplement: Attachment — Submitted filename: Response to Reviewers.docx [file pone.0327769.s002.docx]

# Response to Reviewers’ Comments

We thank the reviewers and editor for their thorough evaluation of our manuscript. We have carefully addressed each comment, as detailed below. All changes in the revised manuscript are indicated with line numbers, original text, and revised text for clarity.

## Reviewer #1

**Comment 1:** “The overall formatting of the manuscript is poor, and the English expression is awkward and unnatural in places.”

**Response:** Thank you for highlighting these issues. We have thoroughly proofread and revised the manuscript to improve its formatting and language clarity. All sections have been checked for proper structure (e.g., adding missing spaces in headings) and the text has been edited for more natural phrasing. Below we provide examples of these corrections:

**Changes in Manuscript (Line 19-21 on page 2):** Original: “As illustrated in Fig 1, the Steemann one-arm pantograph, which is widely adopted in subway systems, employs two sliding strips on its upper assembly. ” → **Revised:** “The widely employed Steemann single-arm pantograph (Fig 1) incorporates two interchangeable carbon sliding strips in its upper structure.”

Extensive revisions to the English expression have been implemented across the entire manuscript. Given that these revisions were pervasive and impacted every paragraph significantly, all paragraphs are highlighted in the submitted Marked-up Copy.docx.

These revisions (and similar edits throughout the paper) have greatly improved readability and adherence to PLOS ONE formatting standards.

**Comment 2:** “This paper is an engineering-focused work, but the literature review is inadequate. It should focus more on studies related to Remaining Useful Life prediction for the Subway Pantograph Sliding Strips, providing a more relevant context.”

**Response:** We appreciate this suggestion. We have expanded our literature review to more directly discuss prior studies on pantograph sliding strip wear and prognostics, thus providing a stronger context for our work. In particular, we now explicitly reference several relevant studies and clarify the gap in remaining useful life (RUL) prediction for pantograph strips. **The revised text (Lines 17-24 on page 5, Lines 1-20 on page 6) ：**“Existing research on pantograph strip wear has primarily focused on predicting strip thickness or classifying its condition, rather than directly estimating the RUL. Kuźnar et al. [1] utilized artificial neural networks to forecast strip thickness with less than 12% error. Xu et al. [2] achieved 97.3% accuracy in wear trend fitting using support vector regression, while Trilla et al. [3] reduced thickness prediction uncertainty to 1.39 mm by employing a time delay neural network ensemble that integrates seasonal variables. Classification methodologies have also been explored, with Kuźnar et al. [4] and Kuźnar and Lorenc [5] demonstrating high accuracy in categorizing strip conditions.

The existing literature exhibits a dearth of studies aimed at predicting the RUL of pantograph sliding strips. To date, only two notable contributions have been identified in this domain. Guan et al. [6] proposed a Wiener process model tailored to address the challenge posed by unevenly spaced wear data in carbon contact strips of railway pantographs. Their methodology integrated the Wiener process with a weighted linear regression model for unevenly spaced intervals, effectively predicting RUL using authentic operational data from the Guangzhou Metro. Conversely, Li et al. [7] developed a reliability-centered framework for predicting RUL by investigating wear patterns under varying contact pressure fluctuations. Through experiments on sliding electrical contacts, they formulated both a longevity prediction model and a probability distribution model for the phase of stable wear.

Despite their potential, both methods exhibit notable limitations that hinder their practical application. The statistical assumptions underlying these models often fail to adequately capture the complexities of real-world scenarios, and they do not seamlessly integrate with current maintenance planning systems. This disconnect between theoretical frameworks and practical needs substantially diminishes their utility for informing maintenance decisions. Moreover, the accuracy of RUL predictions is highly sensitive to the precision of thickness measurements, posing a significant challenge. A mere 1 mm discrepancy in thickness prediction can result in a 33-day deviation in RUL estimation based on typical wear rates. Such considerable inaccuracies could lead to premature replacements or, even worse, unforeseen failures that jeopardize service reliability.”

The above revisions incorporate the most relevant currently available research focused on sliding strip remaining useful life prediction. This provides essential background, clarifies the limitations of existing studies, and highlights the novelty of our current work

**Comment 3:** “Real-world data is crucial for RUL prediction tasks and is highly valuable. Therefore, a more detailed description of the dataset’s characteristics is necessary to strengthen the paper’s foundation.”

**Response:** We agree and have added a more comprehensive description of the dataset used in our study. The manuscript now clearly describes the source, scope, and key characteristics of the data from Chongqing Metro Line 6. We specify the time span of data collection, the number of pantograph strips monitored, and have conducted statistical analyses of the data's monotonicity and random distribution characteristics. These details reinforce the real-world basis of our work and help readers understand the data context. We have added a subsection describing the data characteristics (**from page 10, line 8 to page 13, line 7 of the revised manuscript**), the specific content of which is as follows:

## **Empirical Analysis of Wear Monotonicity Characteristics**

The pantograph sliding strips inevitably experience thinning over time due to irreversible material loss from wear. Our wear analysis corroborates this trend, with data indicating either a steady decrease or constancy in strip thickness. As illustrated in Fig 4, the wear trajectories for twenty strips consistently demonstrate a decline in thickness, although minor deviations may arise from measurement errors. This consistent wear pattern represents a critical aspect that must be incorporated into any robust degradation model [29].

Fig 4. displays twenty typical wear trajectories of individual pantograph sliding strips, demonstrating a consistent decrease in thickness over time.

## Goodness-of-Fit Test for Wear Increments

The task at hand involves the selection of an appropriate probability distribution for these increments. Two potential distributions, namely Gamma and Inverse Gaussian (IG), were evaluated using Anderson–Darling (AD) goodness of fit tests [30]. Each strip's positive wear increments were analyzed by fitting both a Gamma distribution and an IG distribution. Subsequently, the AD test was employed to compare the observed increment data with the cumulative distribution function (CDF) of each fitted distribution. The null hypothesis posited that the increments in the strip follow the specified distribution. If, at a significance level of 5%, the AD test indicated no statistically significant deviation between the empirical data and the fitted CDF, the distribution was considered a suitable model for the strip's increments, demonstrating a successful outcome of the goodness of fit test for that distribution. Conversely, if the AD test detected a significant deviation at the 5% level, the distribution was rejected as an inadequate fit for that strip.

Table 1 displays the goodness-of-fit outcomes for wear increment data under two distributional assumptions. The Gamma distribution notably fits the wear increment data well across the entire fleet. Individual train pass rates for the Gamma distribution range from approximately 89% to 100%, with an overall 97.9% of the 1,120 increment series showing no significant deviation from the fitted Gamma model (Column 3 in Table 1). Specifically, only 18 series were deemed incongruent with the Gamma distribution by the Anderson-Darling (AD) test. Almost all trains adhered to the Gamma distribution, with 17 out of 20 trains having at least 95% of their strips passing the Gamma test; the one exception still achieved around 89%. This strong support highlights the excellent fit of wear increments with the Gamma distribution in nearly all cases.

Table 1 displays the goodness-of-fit outcomes for Gamma and IG increment distributions across different train datasets. The summary includes the number of wear increment series (n) examined and the percentage of series that satisfied the Anderson–Darling test for each distributional assumption.

| Train Number | **n** | **Gamma distribution pass (%)** | **Inverse** Gaussian **distribution pass (%)** |
| --- | --- | --- | --- |
| 1 | 44 | 90.9 | 90.9 |
| 2 | 46 | 97.8 | 95.7 |
| 3 | 44 | 100 | 95.5 |
| 4 | 37 | 89.2 | 83.8 |
| 5 | 31 | 90.3 | 83.9 |
| 6 | 40 | 95 | 82.5 |
| 7 | 34 | 100 | 97.1 |
| 8 | 40 | 100 | 100 |
| 9 | 52 | 100 | 100 |
| 10 | 78 | 96.2 | 96.2 |
| 11 | 79 | 100 | 100 |
| 12 | 76 | 100 | 94.7 |
| 13 | 42 | 100 | 100 |
| 14 | 75 | 100 | 100 |
| 15 | 76 | 98.7 | 97.4 |
| 16 | 72 | 100 | 100 |
| 17 | 79 | 100 | 100 |
| 18 | 73 | 100 | 100 |
| 19 | 65 | 100 | 98.5 |
| 20 | 37 | 100 | 100 |
| Overall | 112020 | 97.9 | 95.8 |

While the Inverse Gaussian distribution achieved an impressive overall pass rate of 95.8%, its consistency across the entire fleet was somewhat lower. Individual train pass rates varied from 83% to 100% under this model, with 38 series showing significant deviations, approximately double the outliers compared to the Gamma distribution. It is notable that some trains, which had perfect pass rates under the Gamma distribution, encountered failures in one or two instances when tested using the Inverse Gaussian model. This highlights the superior ability of the Gamma distribution to capture the full range of observed wear increment patterns.

After comprehensive analysis, the Gamma process emerges as the optimal stochastic model for sliding-strip wear. Among the three processes evaluated (Wiener, Gamma, and IG), only the Gamma and IG processes exhibit consistent sign increments, leading to monotonically decreasing paths. Although the Wiener process is mathematically tractable, it may yield unrealistic thickness fluctuations when unrestricted. Moreover, the wear increment distribution associated with the Gamma distribution, boasting a 97.9% success rate, aligns both theoretically and empirically with models of sliding strip degradation.

This expanded description ensures that the data foundation for our RUL prediction is transparent and well-understood.

**Comment 4:** “The decision to use the Gamma process instead of the Wiener process is not well justified. The Wiener process is also widely used in RUL prediction tasks, as seen in Reliability Engineering & System Safety (DOI: 10.1016/j.ress.2024.110014). A discussion on why the Gamma process was chosen over the Wiener process should be included.”

**Response:** We appreciate this comment and have strengthened the justification for selecting a Gamma process model. In the revised manuscript, we explicitly contrast the Gamma process with the Wiener process and provide reasons for our choice. We note that Wiener-process-based models are indeed popular in RUL prediction (e.g., Guan et al., 2020 applied a Wiener process to railway contact system wear data).

In the "**Stochastic Degradation Model Selection and Validation**" section, we have now discussed in detail the reasons for selecting the Gamma process. Firstly, based on a comprehensive analysis of existing literature, we emphasize that the Wiener process allows bidirectional stochastic fluctuations around a trend (suitable for non-monotonic degradation) unless additional constraints are imposed, whereas pantograph strip wear is monotonic (cumulative wear only increases). Because a sliding strip’s thickness only decreases over time, a Gamma process—which inherently models monotonically increasing degradation—better fits the physical reality. Additionally, we validated this choice based on the characteristics of our data; specifically, the fact that the increments in wear align more closely with a Gamma distribution further supported our decision not to select the Wiener process or the Inverse Gaussian process.

This added discussion directly addresses the reviewer’s concern and cites the suggested reference context, making our methodological choice clear and well-founded.

**Comment 5:** “The applicability of the proposed method is unclear. For example, similar operational and maintenance requirements exist in high-speed rail systems (see Vehicle System Dynamics, DOI: 10.1080/00423114.2024.2369718). It would be beneficial to explore whether the proposed method could be applied to such systems.”

**Response:** We agree that discussing the broader applicability of our approach adds value. In the revised manuscript, we have included a statement in the Discussion noting that our Gamma process-based RUL prediction framework could potentially be extended beyond subway systems. We specifically mention that high-speed railway systems have analogous maintenance challenges (e.g., pantograph wear under higher speeds) and that our method could be adapted to such contexts in future work. We have cited the suggested reference to acknowledge similar needs in the high-speed rail domain.

**The specific revisions are detailed below:**

The methodology under consideration is easily transferable to the examination of analogous transportation systems, like high-speed rail and other transit networks, as evidenced in a recent investigation on gearbox functionality [46]. These systems exhibit fundamental resemblances to pantograph strips, such as uniform wear patterns under different operational loads, standardized inspection procedures, and established safety protocols. Tailoring the model to these alternative systems mainly involves adjusting the data sampling methodology to accommodate distinct operational traits and validate the training data's precision, along with refining pertinent parameters instead of necessitating substantial system restructuring.

By including this forward-looking statement, we clarify the potential of our work to inform maintenance practices in high-speed rail and other related systems.

**Comment 6:** “The resolution of all images in the manuscript should be improved to ensure clarity and readability.”

**Response:** We have addressed this concern by replacing **all figures** in the manuscript with higher-resolution versions. The images are now provided at sufficient resolution (at least 300 DPI) to ensure clarity of details (especially for plots and diagrams). This improvement will enhance readability and allow readers to interpret the figures without difficulty.

**Changes:** All figure files have been updated to high-resolution images. (There are changes in the figure captions or numbering and the image quality has been improved.)

## For example, the lines in the previous Fig 8 and 9 were not very clear, but they have been clarified in the revised manuscript. We are confident that all figures now meet the required clarity standards.

## Reviewer #2

**Comment 1:** “The Abstract could benefit from a clearer emphasis on the practical significance of this research. Highlighting the engineering context and potential real-world applications would help readers better understand the value of the proposed method.”

**Response:** We have revised the Abstract to underscore the practical engineering significance of our work. The updated Abstract now explicitly mentions the maintenance context and the real-world benefits achieved by our method. By highlighting the economic and operational impact of our RUL prediction framework, we provide readers with a clearer understanding of its value in practice.

**The revised abstract is as follows:**

“The progressive wear of pantograph sliding strips on metro trains necessitates timely replacement to ensure safe and reliable operations. This study proposes an adaptive, data-driven framework for predicting the remaining useful life (RUL) of these components, leveraging operational data from Chongqing Metro Line 6. A Gamma-process model is employed to capture the wear behavior under real-world operating conditions, integrating historical records and new observations through Bayesian inference. Markov chain Monte Carlo (MCMC) sampling is then applied to solve the posterior distribution, with three parameter-estimation approaches compared and the model's predictive accuracy evaluated across different life-cycle stages. The results demonstrate that incorporating prior knowledge significantly improves prediction accuracy. To showcase practical utility, the study devises a maintenance-scheduling strategy that integrates RUL forecasts with regular vehicle-maintenance intervals, thereby extending service life and reducing costs. Validated using real-world data, the proposed methodology offers a pragmatic tool for predictive maintenance in metro systems and can be adapted to similar engineering applications”

This revised Abstract now clearly communicates the real-world applications and benefits of our research up front, as requested.

**Comment 2:** “While the authors have provided a thorough review of current research, it would be beneficial to more explicitly summarize the existing research gaps before introducing the contributions and novelty of this work. Additionally, expanding the discussion to include emerging trends in machine learning and signal processing—particularly in industrial applications—could strengthen the manuscript. For example, exploring connections to recent advancements in areas such as digital twin methodology for vibration-based monitoring and prediction of gear wear, review of vibration-based gear wear monitoring and prediction techniques, and neuro-fuzzy system-guided cross-modal zero-sample diagnostic framework using multi-source heterogeneous non-contact sensing data could provide valuable context and demonstrate the broader relevance of this research.”

**Response:** We appreciate this detailed suggestion. We have modified the end of our Introduction to clearly summarize the research gaps and to incorporate the contents of emerging trends in prognostics and machine learning for industrial applications in Discussion.

**The following is the revised summary of existing research gaps:**

“Despite their potential, both methods exhibit notable limitations that hinder their practical application. The statistical assumptions underlying these models often fail to adequately capture the complexities of real-world scenarios, and they do not seamlessly integrate with current maintenance planning systems. This disconnect between theoretical frameworks and practical needs substantially diminishes their utility for informing maintenance decisions. Moreover, the accuracy of RUL predictions is highly sensitive to the precision of thickness measurements, posing a significant challenge. A mere 1 mm discrepancy in thickness prediction can result in a 33-day deviation in RUL estimation based on typical wear rates. Such considerable inaccuracies could lead to premature replacements or, even worse, unforeseen failures that jeopardize service reliability.”

**In the discussion section, we have added further discussion points, as detailed below:**

Recent technological advancements offer significant opportunities to enhance and broaden our approach to predicting RUL. Digital twin technology allows for the creation of real-time virtual replicas of physical assets, enabling detailed simulation and predictive analysis. In the context of pantograph systems, digital twins can incorporate the Gamma process model to simulate wear progression by adjusting parameters based on operational variables. Additionally, the integration of diagnostic frameworks that combine data from various sensors, such as vibration signals, acoustic emissions, temperature readings, and electrical data, is essential for comprehensive equipment health evaluations. By amalgamating data from multiple sensor types, the proposed framework can identify degradation patterns that might be missed by single-sensor methodologies.

With these additions, the Introduction now clearly delineates the gap and situates our work within both the specific pantograph context. And the discussion introduce the wider landscape of modern prognostic techniques.

**Comment 3:** “The resolution of the figures in the manuscript could be improved to ensure clarity and enhance the overall presentation of the results.”

**Response:** We have updated **all figures** to high-resolution versions to improve clarity, as also noted in our response to Reviewer #1. The plots, diagrams, and images in the revised manuscript are now much clearer (at the recommended resolution), which significantly enhances the presentation of our results.

**Changes:** All figure images have been replaced with higher-resolution files;

The improved figure quality will ensure that readers can easily see all details (for example, small text and markers in graphs are now crisp and legible).

**Comment 4:** “There are occasional grammatical errors throughout the manuscript. A thorough proofreading to address these issues would improve the readability and professionalism of the paper.”

**Response:** We have thoroughly proofread the manuscript and corrected all identified grammatical and typographical errors. This includes fixing article usage, verb tenses, pluralizations, and punctuation to ensure the text reads smoothly and professionally.

All such issues have been addressed in the revised manuscript. We are confident that the paper’s language is now clear and correct throughout.

**Comment 5:** “Including a section on potential future research directions at the end of the Conclusion would provide a forward-looking perspective and inspire further exploration in this field.”

**Response:** We have added a dedicated discussion of future research directions at the end of the Conclusion section. In the revised Conclusion:”**Future research should prioritize enhancing the framework's capabilities and validating its effectiveness across various wear mechanisms and operational cycles, such as high-speed rail gearboxes and other transportation assets. The integration of continuous Internet of Things sensor data could facilitate real-time updates on Remaining Useful Life through efficient online Bayesian algorithms. Hybrid models that merge Gamma processes with deep learning could adeptly capture nonlinear degradation patterns while maintaining interpretability. Enhancing computational efficiency via advanced sampling methods or parallel processing would support real-time fleet-wide implementation. By optimizing maintenance activities for critical components, overall lifecycle expenses could be reduced. These advancements have the potential to elevate the prototype into a comprehensive predictive maintenance platform suitable for deployment in urban and high-speed rail networks**.”

This explicit “Future Directions” section at the end of the paper provides the recommended forward-looking perspective and emphasizes how our research can be expanded in subsequent studies.

## Additional Revisions (Editorial Requirements)

In addition to the above reviewer comments, we have made the following editorially requested changes to ensure compliance with PLOS ONE guidelines:

**Role of Funder Statement:** As requested, we have included the Role of Funder Statement in the Cover Letter. The specific content is as follows:The funders had no role in study design, data collection and analysis, decision to publish, or preparation of the manuscript.

**Funding Statement Update:** We have removed the funding information from the Acknowledgments section and updated the Funding Statement as per the journal’s requirements. We have included the revised Funding Statement information in the Cover Letter; the specific information is: "This work is supported by the Science Technology Research Program of Chongqing Municipal Education Commission (Grant No. KJQN202203405, KJQN202303402, KJQN202203417)." This clarifies the role of the funding source. (The Acknowledgments now only thank the Chongqing Rail Transit Group for data support).

**Figure 15 Reference:** We noticed (as pointed out by the editor) that **Figure 15** was not explicitly cited in the original text. We have added a sentence in the Results section to reference Figure 15 and describe its content.

**Changes in Manuscript (around Line 6 on page 36 of the previous version):** Original text did not mention Figure 15. → Revised to include: “The importance of incorporating prior information is highlighted in the evaluation of prediction accuracy, as demonstrated in Fig 15.” (Now Line 3-4 on page 38 of the revised manuscript).

This insertion ensures that Fig 15 is properly cited and discussed in the text, allowing readers to understand its role in the analysis.

**PLOS ONE Formatting:** We have reviewed the manuscript to comply with all PLOS ONE formatting requirements. This includes correct heading formats (as shown in our correction for the Introduction heading), proper in-text figure and table citations (all figures including Fig 15 are now referenced in order), and adherence to the journal’s style for units and abbreviations. .

We believe that the revised manuscript addresses all the reviewers’ comments and editorial requests. The content is now clearer, more contextualized with relevant literature, and formatted correctly. We thank the reviewers and editors again for their valuable feedback, which has helped us improve the quality and clarity of our work. Please let us know if any further adjustments are needed.
